# The contribution of single-cell analysis of acute leukemia in the therapeutic strategy

**DOI:** 10.1186/s40364-021-00300-0

**Published:** 2021-06-27

**Authors:** Lamia Madaci, Julien Colle, Geoffroy Venton, Laure Farnault, Béatrice Loriod, Régis Costello

**Affiliations:** 1 Laboratoire TAGC/INSERM UMR 1090, Parc Scientifique de Luminy case 928, 163, Avenue de Luminy, Cedex 09, 13288 Marseille, France; 2 Service d’Hématologie et Thérapie Cellulaire, Hôpital La Conception, Assistance Publique des Hôpitaux de Marseille, 147 boulevard Baille, 13005 Marseille, France; 3 TGML-TAGC/INSERM UMR1090 Parc Scientifique de Luminy case 928, 163, avenue de Luminy, Cedex 09, 13288 Marseille, France

**Keywords:** Acute myeloid leukemia, Single cell, Tumor heterogeneity, Clonal evolution, Drug resistance, Targeted therapy, Leukemia stem cell, Next generation sequencing

## Abstract

After decades during which the treatment of acute myeloblastic leukemia was limited to variations around a skeleton of cytarabine/anthracycline, targeted therapies appeared. These therapies, first based on monoclonal antibodies, also rely on specific inhibitors of various molecular abnormalities. A significant but modest prognosis improvement has been observed thanks to these new treatments that are limited by a high rate of relapse, due to the intrinsic chemo and immune-resistance of leukemia stem cell, together with the acquisition of these resistances by clonal evolution. Relapses are also influenced by the equilibrium between the pro or anti-tumor signals from the bone marrow stromal microenvironment and immune effectors. What should be the place of the targeted therapeutic options in light of the tumor heterogeneity inherent to leukemia and the clonal drift of which this type of tumor is capable? Novel approaches by single cell analysis and next generation sequencing precisely define clonal heterogeneity and evolution, leading to a personalized and time variable adapted treatment. Indeed, the evolution of leukemia, either spontaneous or under therapy selection pressure, is a very complex phenomenon. The model of linear evolution is to be forgotten because single cell analysis of samples at diagnosis and at relapse show that tumor escape to therapy occurs from ancestral as well as terminal clones. The determination by the single cell technique of the trajectories of the different tumor sub-populations allows the identification of clones that accumulate factors of resistance to chemo/immunotherapy (“pan-resistant clones”), making possible to choose the combinatorial agents most likely to eradicate these cells. In addition, the single cell technique identifies the nature of each cell and can analyze, on the same sample, both the tumor cells and their environment. It is thus possible to evaluate the populations of immune effectors (T-lymphocytes, natural killer cells) for the leukemia stress-induced alteration of their functions. Finally, the single cells techniques are an invaluable tool for evaluation of the measurable residual disease since not only able to quantify but also to determine the most appropriate treatment according to the sensitivity profile to immuno-chemotherapy of remaining leukemic cells.

## Background

After decades during which the treatment of acute myeloblastic leukemia (AML) was based on variants of the classic “7 + 3” cytarabine/daunorubicin association (apart from the specific treatment of promyelocytic AML), progresses in molecular biology by next generation sequencing (NGS) have made possible to discover numerous drugable mutations leading to the notion of targeted therapies.

The NGS techniques, also called “massively parallel sequencing” or “deep sequencing”, can analyze millions of DNA or RNA fragments in parallel. The first step of NGS is the random DNA fragmentation followed by binding to specific small sequences, followed by amplification of this bank using clonal amplification and PCR methods, then the sequencing is performed, usually by the sequencing synthesis (SBS) technique (for a review, [1]). This technology allows sequencing a whole exome (22,000 coding genes) within a single day, while the classical Sanger technique would require months or years. The NGS has many advantages over conventional techniques. Firstly, it allows the detection of all mutations but also single nucleotide polymorphisms (SNIPs) in a given cell population without the need to define a priori the target genes. Then, this technique has a high sensibility allowing sequencing even very small cellular subpopulations. Finally, the cost of whole genome sequencing is admittedly expensive, but much cheaper (less than 1000 dollars) than previous technologies. Nonetheless, NGS technology has some disadvantages. First, NGS requires a dedicated technologic platform with potent bioinformatics systems, since raw data cannot be directly used due to their huge amount (mega-datasets). Along the technological pitfall, the correct assembly of repeated region of DNA is problematic, as the detection of short deletions. Finally, a high number of abnormalities or variations (mutations, SNIPs) can be identified which clinical significance is unknown, thus remaining in the field of research but not useful in routine clinical practice. Despite these drawbacks, the availability of NGS has greatly advanced our knowledge in oncology. Combined with single cell analysis, this approach is illustrated by the Cancer Moonshot Initiative project led by the National Cancer Institute. The goal of this project is to obtain a multi-parametric atlas at the single cell level of 11 types of cancers, including the identification of pre-cancerous stages, established cancer and then the evolution with metastasis and resistance/escape to therapeutic. The ultimate goals of this project is to identify predictive markers, to understand the key features of previously described transition states, and the to identify relevant therapeutic targets in order to elaborate specific treatment strategy at the individual level, *i.e* precision-medicine treatment designed to fit with both the tumor and the patient [2].

While bulk tumor cell analysis has allowed great advances in cancer treatment, the requirement for more precise analysis at unique cell level is more and more necessary for a comprehensive assessment of tumor biology and, from a clinical point of view, risk stratification. Different techniques are at our disposal (for a review, [3]), from flow-cytometry to the transformational technology of single-cell RNA sequencing (scRNA-seq) allowed by the developments of NGS. Cell-by-cell analysis is the hallmark of flow cytometry. While this technique has evolved considerably in recent years, the number of labeled antibodies that can be used for a single sample is limited by the auto-fluorescence of the cells and by the fluorescent dye spectral overlap. While some instruments can analyze up to 50 parameters simultaneously, most recent machines do not exceed 20 fluorescence detectors [4]. Mass spectrometry techniques make it possible to increase the number of parameters analyzed, up to 120 in the most recent publications, which have improved our knowledge of certain rare populations such as leukemia stem cells (LSCs) [5, 6]. Even the analysis of the expression of 120 molecules is nevertheless far from the thousands of genes expressed by each cell. The scRNA-seq techniques determine the expression, in a semi-quantitative way, of all the RNAs of each cell, on samples of about tens of thousands of cells. Computer analysis of the data enables cell populations to be determined according to the degree of similarity of the gene expression pattern. Given the cost of this type of experiment but also the possible variations from one experiment to another, techniques have been developed to allow several different samples to be analyzed simultaneously, for example the cell hashing. This technique uses antibodies that recognize ubiquitous antigens. Then the addition of a different barcode to each antibody allows, after independent cell labelling, recognizing each cell of each sample once they have been mixed [7]. Several different samples (4 to 8) can be analyzed at the same time, with the limitation of the number of cells analyzed per sample. The single cell technique can be associated with the direct recognition of cell subpopulations by the cellular indexing of transcriptomic and epitopes by sequencing (CITE-seq) technique [8], based on the principle of labelling by an antibody coupled to a barcode. Other techniques allow the analysis of genomic sequences, chromatin accessibility, DNA methylation, histones and chromosome conformation. These different techniques can be combined for multimodal analyses [3]. The application of these techniques to AMLs has led to interesting results with numerous physiopathological, prognostic and probably therapeutic benefits.

## The results obtained by targeted therapies

If we consider the new marketing authorizations for AML drugs, most of them belong to the class of targeted therapies: midostaurine and gilteritinib (anti- Fms-like tyrosine kinase 3 [FLT3)), enasidenib (anti-isocitrate deshydrogenase [IDH]2), ivosidenib (anti-IDH1), venetoclax (anti- B-cell Lymphoma 2 [BCL2]), glasdegib (Hedgehog [Hh] inhibitor). From a simple diagnostic and prognostic tool, the large-scale identification of molecular abnormalities in AMLs [9], made possible by the high throughput of NGS, has made them therapeutic targets (for a non-exhaustive overview, see Table 1). But do the results obtained live up to our expectations? We will limit our discussion to the results obtained by the inhibitors of FLT3, Hh, IDH1/ IDH2 and BCL-2. We will differentiate and discuss separately the monotherapy trials, which will give us the most accurate information about how the drugs work and fail.

**Table 1:** a non-exhaustive overview of most frequent mutations in AML, with their functional overlap, examples of targeted therapy and prognosis value (the effect of mutation associations on the prognosis has not been developed). *: double CEBPA mutation. NS: not significant

Functional Mutations	Genes	Functional Overlaps	Targeted Treatment	Prognostic Value
Signal transduction and oncogenes	FLT3 NRAS KRAS KIT	Transcription factors	Midostaurin Gilteritinib Farnesyl transferase Tyrosine kinase inhibitors	Poor Poor NS Poor
Splicing mutations	SF3B1 ZRSR2 U2AF1 SRSF2	Epigenetic modifiers	H3B-8800 GSK3326595 Gilteritinib	NS Poor Poor Poor
Transcription factors	RUNX1 CEBPA GATA2 BCOR BCORL	Oncogenes Epigenetic modifiers	Sorafenib tosylate Inhibitors of lysine specific demethylase 1 (LSD1) JAK/STAT inhibitors	Favorable Good* Poor Poor
Epigenetic modifiers	BCOR BCORL SRSF2 DNMT3A IDH1 IDH2 TET2 ASXL1 EZH2	Splicing Transcription Tumor suppressor Chromatin modifiers	AG-120 AG-221 BI 836858 Bromodomain inhibitors	Poor Poor Poor Poor Favorable Poor Poor Poor
Chromatin modifiers	ASXL1 EZH2 Cohesin	Epigenetic Tumor suppressor	mTORC1/mTORC2 inhibitor Bromodomain inhibitors	Poor Poor NS
Tumor suppressors	TET2 TP53 WT1	Epigenetic Chromatin modifiers	BI 836858 pevonedistat Entospletinib TP-0903	Poor Poor Poor
Licensing mutations	NPM1		entospletinib	Favorable

### Inhibition of IDH1/IDH2

Recurrent mutations of the enzymes IDH1 and IDH2 are present in 15 to 25% of AML cases. The normal enzyme allows the conversion of isocitrate to alpha-ketoglutatrate (KG) which plays an important role in the regulation of oxidative stress. On the other hand, mutated IDH1/IDH2 enzymes convert KG into R-2-hydroxyglutarate (2HG) which modify histone demethylation (one of the mechanisms involved in leukemogenesis) by inhibiting the Jumonji C domaine-containing demethylase (JMJD2A) and the ten-eleven translocation 2 (TET2) molecule. These mutations often occur in association with nucleophosmin (NPM1) and DNA-MethylTransferase 3 (DNMT3) mutations. When associated with an NPM1 mutation, the prognosis is significantly improved. This notion of co-association of mutations is an important element to take into account, which may already begin to pose a problem in the concept of targeted therapy; what molecular anomaly should be targeted when there is an association? Certainly, it is possible to think the solution comes from clinical trials. But in this case, given the multiple possible associations, it will be very difficult to include enough patients in a clinical trial to derive statistically valid results for each possible association. In the Phase I trial of the IDH1 inhibitor ivosidenib, a complete remission rate (CR) of almost 22% was achieved, but with a median duration of response of only 6.5 months and a median overall survival (OS) under 9 months, thus demonstrating rapid tumor escape mechanisms [10, 11]. With the IDH2 inhibitor enasidenib, a CR rate of almost 20% was achieved, but with a median duration of response still under 6 months and a median OS under 9 months. How can we try to explain these results, which are certainly interesting but insufficient in view of the brevity of the response and the absence of a significant cure rate? Is it a lack of efficacy of the drug in eradicating IDH mutated cells? The ivosidenid allows the disappearance of the IDH1 mutation in 21% of cases, with a median response rate that in this case increases to more than 15 months, without being able to attain AML cure because the mutated clones ultimately reappear. Nevertheless, the notion of negativity of measurable residual disease (MRD) detected in molecular biology must be evaluated with caution, as it seems that a number of MRD-negative patients present a contingent of mutant IDH1 cells only detected by specific antibodies [12]. The simplest hypothesis consists in considering that a fraction of IDH1 mutated cells are not sensitive to the drug, by diverse mechanisms that could possibly be identified in the total population or by more sophisticated “single cell” analysis techniques. Certain mechanisms leading the IDH2 inhibitor enasidenib to induce its own inactivation can be hypothesized. Enasidenib at clinically useful doses induces up to a three-fold increase of the cytochrome CYP3A [13]. CYP3A is involved in N-dealkylation [14], this mechanism being in humans involved in the degradation of enasidenib [15]. The article by Quek et al. [16] analyzes the clonal evolution of patients treated with enasidenib. Firstly, the hypothesis of new mutations not sensitive to enasidenib, as observed with tyrosine kinase inhibitors in chronic myeloid leukemia (CML), is not retained by the authors because no new IDH2 mutations were identified. This mechanism has nevertheless already been described to explain relapses/resistance to enasidenib, whether by trans or cis dimer-interface mutations [17]. The mechanism of action retained for enasidenib is not the eradication of the mutant IDH2 clones but the terminal differentiation of part of the mutant clones, allowing a resumption of normal hematopoiesis. In addition, other clones with mutations other than IDH2 may contribute to the relapse, either because they exist at an undetectable level at the time of diagnosis, or by their de novo appearance [16]. Indeed, IDH mutations are considered as foundational but are often associated with other mutations such of DNMT3, serine/arginine-rich splicing factor 2 (SRSF2), NPM1, Additional SeX combs Like 1 (ASXL1) and Runt-related transcription factor 1 (RUNX1) in particular, which may contribute to relapse through clonal or sub-clonal evolutionary mechanisms. Nevertheless, DiNardo’s seminal paper [10] did not find a correlation between pre-existing single gene mutations, including Neuroblastoma Rat Sarcoma (NRAS), and the response rate. Nonetheless in refractory patients an enrichment of mutations involving the metabolic pathway of tyrosine kinase receptors was observed. In addition, the mean number of mutations was 1.8/patient in the responder group vs. 2.6/patient in the non-responder group, thus supporting escape mechanisms related to mutations not sensitive to IDH1 inhibition. Can these results be improved by a combined approach? DiNardo et al. (Abstract 560, ASH Annual Meeting 2018) treated 154 patients with IDH1 or IDH2 mutations with a classic 3 + 7 regimen (daunorubicin or idarubicin) combined with ivosidenib or enasidenib, with consolidation and continuation of IDH inhibitors. The overall response rate (CR + CR with incomplete count recovery [CRi]) was 80%, with a CR rate of 91% for patients with primary AMLs, and 53% for patients with secondary AMLs. Several other studies are in progress to test the therapeutic gain of a combination of chemotherapy/demethylating agents with anti-IDH, the results of which are pending. (https://clinicaltrials.gov/ct2/show/NCT03173248, https://clinicaltrials.gov/ct2/show/NCT02677922).

### Hedgehog inhibition: glasdegib

This oral Hh pathway inhibitor targets the smoothened compound (SMO), which is abnormally present in AMLs and induces signaling via the glioma-associated oncogene homolog (GLI1). Numerous in vitro studies underline the potential value of inhibiting this signaling pathway in AMLs, notably through a chemo-sensitizing effect on LSCs [18] known to be resistant to conventional drugs [19]. In a Phase I trial, 28 patients with AML were treated with glasdegib [20]. The results, although interesting, were not spectacular: an effect was observed in 16 of the 28 patients, with 1 CR, 4 CRi, 4 patients with a minor response and 7 patients with stable disease. Many other studies have investigated the combination of glasdegib/chemotherapy. Cortes et al. compared low-dose cytarabine to cytarabine/glasdegib in patients with AML or high-risk myelodysplastic syndrome (MDS) [21]. The CR rate was 17% in the cytarabine/glasdegib arm vs. 2.3% in the cytarabine arm alone, with an OS of 8.8 months vs. 4.9 months respectively. In the absence of a randomized trial, it nevertheless appears that by analyzing the literature data and by simulating treatment comparisons, the glasdegib/cytarabine combination is superior to azacytidine or decitabine treatments [22]. In another single-arm trial, glasdegib was combined with conventional “7 + 3” cytarabine + daunorubicin chemotherapy, with a CR rate of 46.4% in patients primarily over 55 years of age [23]. This CR rate is not particularly favorable, the authors having set a goal of exceeding a CR rate of 54%, but the observation of a plateau of relapse-free survival (RFS) at 24 months for about 40% of patients suggests an eradicating effect on LSCs. However, it should be noted that in this study no correlation between clinical response and mutations in the 12 genes analyzed (CCAAT/enhancer-binding protein alpha [CEBPA], DNMT3A, FLT3, FLT3- internal tandem duplication [ITD], IDH1, IDH2, KIT, KRAS, NPM1, NRAS, RUNX1, TET2, Wilm’s tumor 1 [WT1]) could be demonstrated. In MDS refractory to hypomethylating agents, glasdegib monotherapy gives a response in only 6% of patients (2 out of 35), with nevertheless 56% of patients showing stable disease for an OS of these patients of 20.6 months [24].

### Inhibition of FLT3 (midostaurin, gilteritinib)

FLT3 is a class III receptor tyrosine kinase, and is the most common mutation found in AMLs, justifying the interest of targeted therapies that would be applicable in about 30% of patients [25]. The seminal article of Stone et al. [26] in FLT3 mutated patients compared standard daunorubicin plus cytarabine induction and consolidation therapy with high-dose cytarabine with or without midostaurin. The results in terms of CR are not significant, 59% in the midostaurin group vs. 54% in the placebo group, but with significantly improved OS and event-free survival (EFS) in the midostaurin arm. It should be noted that midostaurin has several targets outside FLT3: c-kit, Vascular Endothelial Growth Factor Receptor (VEGFR), Platelet Derived Growth Factor Receptor (PDGFR) and Fibroblast Growth Factor Receptor (FGFR) in particular, which are mutated in some AMLs. This suggests that in some cases an increased efficacy could be due to the existence of several targets. Conversely, the Hh pathway seems to be more often involved in the proliferation of FLT3 mutated AMLs [27], and the double inhibition FLT3/Hh could improve the results obtained with midostaurin. Several other FLT3 inhibitors have been developed, among the most promising being gilteritinib, an inhibitor mainly of FLT3, more incidentally of c-Kit, but also of AXL tyrosine kinase, the blocking of the latter reinforcing the inhibition of FLT3-ITD. Gilteritinib showed efficacy in relapsed/refractory AMLs in the study by Perl et al. [28]. The percentage of CR/CRi patients was 34% in the gilteritinib group vs. 15.3% in the chemotherapy group, with 21.1 and 10.5% CR respectively. While the EFS was 2.8 months in the gilteritinib group vs. 0.7 months in the chemotherapy arm, the survival curves converged at 24 months and there was no survival plateau, clearly demonstrating that gilteritinib monotherapy is an interesting molecule with limited results. Several combination trials of anti-FLT3 antibodies with azacytidine have been conducted [29] without any real benefit having been demonstrated. The mechanisms of resistance to FLT3 inhibitors may be present from the outset in the leukemic population or acquired through selection phenomena. Among the most frequently mentioned mechanisms are mutations at the inhibitor binding site (residue D835 in particular), overexpression of FLT3, or use of alternative signaling pathways (NRAS mutations for example).

### Inhibition of BCL2: venetoclax

For a long time, the simplistic view of an apoptosis defect predominant in lymphoid proliferations (chronic lymphocytic leukemia [CLL] type) and an increase in proliferation as the hallmark of AMLs has prevailed, with the known successes in the use of the BCL-2 inhibitor venetoclax in CLL. Nevertheless, increased BCL-2 expression has been shown to be a poor prognostic factor in AMLs [30]. BCL-2 interferes with pro-apoptotic factors such as Bcl2-associated X protein (BAX), preventing mitochondrial outer membrane permeabilization (MOMP) and thus blocking apoptosis. In a Phase II trial [31], a response to the venetoclax inhibitor was demonstrated with an overall response rate of 19%, with a CR rate of 6 and 13% CRi, with significantly more favorable response rates in IDH1/2 mutated patients with 33% CR/CRi. Conversely, FLT3-ITD and Tyrosine-protein phosphatase non-receptor type 11 (PTPN11) mutations appear to confer resistance to venetoclax [32]. This response to BCL-2 inhibition is undeniably interesting but insufficient. The addition of cytaratbine significantly improves the results with 48% CR/CRi for venetoclax/cytarabine vs. 13% for cytarabine alone, without however resulting in a significant increase in OS (7.2 months for venetoclax/cytarabine vs. 4.1 months for cytarabine alone) [33]. Association with demethylating agents appears more promising with CR/CRi rates of 73% in the azacytidine/venetoclax arm, and median OS not achieved in this arm but > 17.5 months [34], defining a new standard of treatment for AML in subjects > 65 years old and ineligible for intensive chemotherapy [35]. The pathophysiological basis of the efficacy of this association seems to be partly due to the induction of the BH3-only damage (NOXA) protein [36]. Resistance to these therapies is a very rapid dynamic phenomenon with, in particular, gain in FLT3-ITD and loss of TP53 being at the origin of resistance to both ventoclax and chemotherapy [37]. Interestingly, one publication evaluated the response to the venetoclax/azacytidine combination according to the French-American-British (FAB) classification based on blast morphology, which defines among others the AML5 type as monocytic, which turns out to be refractory in 62% of cases [38]. In the same article, the analysis of patients in relapse shows a drift towards a predominantly monocytic profile. This primitive resistance of FAB-AML5 and monocytic selection is explained by the loss of BCL-2 from this subpopulation, in correlation with the maturation towards the monocytic phenotype.

In conclusion, the targeted therapies notably used in monotherapies give interesting results without being spectacular, including little or no “cure”, i.e. sustainable CR. One way to improve these results is the simultaneous use of combination of targeted therapy, as illustrated by the synergistic effect of BCL-2 and FLT-3 co-inhibition [39]. The mechanisms evoked for escape from targeted therapies are obviously multiple, and linked both to the state of the tumor at the beginning of treatment but also to its evolution under treatment, under the possible pressure of targeted therapy +/− conventional chemotherapy +/− adoptive immunotherapy +/− immunity developed by the patient. These different mechanisms can be expressed in different forms. The selection pressure may allow the emergence of clones presenting mutations insensitive to the targeted therapy either appearing de novo by mutation or pre-existing in small numbers and gradually replacing the sensitive cells, the latter hypothesis being favored in mathematical models, at least in CLL in the context of treatment with a Bruton tyrosine kinase (BTK) inhibitor [40]. They may also be clones or cell populations that are capable of using several rescue pathways on the same metabolic pathway or by the use of parallel metabolic pathways. According to these various mechanisms, what means do we have at our disposal to best adapt the treatment to each patient and therefore especially to each tumor? The tumor identity card of AMLs as it is carried out today includes classical cytology data (morphology), flow cytometry phenotyping which allows us to analyze combinatorial surface markers and to define a substantial number of sub-populations, and molecular biology data which allow us to identify, among other things, a rather large (and growing) panel of mutations. Unlike morphological cytology and flow cytometry, which analyze AMLs cell by cell, mutation search techniques are usually performed on bulk populations and give quantitative or semi-quantitative data without identifying, as flow cytometry would do, sub-populations with different mutation profiles. Are these data sufficient to define the best treatment for each patient? To guide it, certainly, but the analysis nevertheless seems insufficient. Let’s imagine an AML for which the molecular diagnosis finds an NPM1 mutation, whose prognosis is modified by the presence of competing mutations of FLT3, DNMT3, NRAS, IDH or PTPN11 for example. The questions that can be asked in this case are multiple; 1) at what level of leukemic differentiation are the mutations (stem cells vs. cells undergoing differentiation), 2) at what level of the clonal hierarchy (truncal mutations vs. branches) are these abnormalities located, 3) at what point are the mutated genes transcribed and in what dynamics (residual RNA or RNA in constant supply)? 4) Are the detected abnormalities grouped on the same cell or are they observed on different cells? (being able to describe co-expression for an unique cell). If, for example, several mutations such as DNMT3, SRSF2, NPM1, ASXL1 and RUNX are found, unless it is considered that these mutations involve all cells, it is impossible to know if the same cell has two or more mutations. At most, if we consider that the mutations are randomly distributed, could we evaluate the co-occurrences by a calculation according to Poisson’s mathematical law. Each of these combinations of expression can lead to a different evolution of AML under treatment, and can be identified in the framework of new single cell technologies.

## Single-cell approaches in leukemia

The single cell approach is of great interest in hematological malignancies, in particular in multiple myeloma, lymphoma and leukemia (for review, [41]). The single-cell approach allows first of all to better define the biology and the heterogeneity of the different blast populations in AMLs [42], normal and leukemia hematopoiesis [43], more accurate prediction of gene mutations, cell classifications, and evolution progression, thereby contributing to risk stratification.

### Deep subtyping of leukemia cell populations and leukemia stem cell

The very notion of LSC is sometimes discussed, nevertheless this population is considered to be at least partly contained the CD34 + CD38- population. It is on this phenotype that the study by Won et al. was based [44], which does not use single cell molecular analysis but classical single cell culture techniques. By comparing the LSCs to the CD34 + CD38+ counterpart, it is shown (on a reduced panel of 84 genes) that there is a decrease in the expression of 27 genes involved in cell activation pathways, in particular concerning the tumor growth factor (TGF), Wbt, FGF, Hh, Notch, IL6ST (interleukin 6 signal transducer) and leukemia inhibitory factor receptor (LIFR) super-families. Another explanation for the quiescence of these LSCs is the decrease in mitochondrial DNA copy number, which, unprotected by histones and closer to the reactive oxygen species (ROS) generation site, is potentially more susceptible to mutations. Sachs et al. [45] identified in a single cell technique a LSCs signature in a mouse model of leukemia that was compared to the transcriptomic profile of CD34 + CD38- stem cells from healthy donors. The similarities of expression in murine LSCs and normal stem cells lead to the hypothesis that the normal transcriptional program is also used for the development of human LSCs. Moreover, by comparing the expression of a set of genes in the 10% of cells expressing them most on normal stem cells vs. LSCs, a signature of 9 LSCs specific genes was demonstrated. This single cell study showed that in LSCs as well as in HSCs, the proliferation and self-renewal functions are separated, possibly explaining the failures of anti-proliferative therapies [45]. Interestingly, Zhu et al. have identified by single cell analysis a master regulator of LSC stemness (in a T-ALL model), that could become a drugable target aiming at LSC eradication [46].

Ultimately, the classical single cell culture and the transcriptomic approach provide us with additional data on the biology of LSCs, with both a decrease in the expression of activation pathways, increased expression of genes involved in self-renewal, separation between proliferation and self-renewal functions, and resistance to chemotherapies while providing possible therapeutic targets aimed at eradicating LSCs (CD36, MYB or SPI1for example).

### Prognostic, risk stratification and evolutionary value

A certain number of AMLs occur during the evolution of an MDS, the evolutionary risk being evaluated by the revised-international prognostic scoring system (R-IPSS) score, which essentially takes into account cytopenias, the percentage of blasts and cytogenetic abnormalities, bearing in mind that NGS techniques have made it possible to highlight the prognostic role of certain mutations [47]. Intuitively and according to these models, which weigh heavily in their scores the percentage of blasts to determine the chances of transformation into AML, the leukemic evolution would be based on these blasts existing at the diagnosis of MDS, whose expansion would lead to AML. The single cell studies challenge this notion by proposing a non-linear progression model. Indeed, the study of a series of patients whose MDS has evolved towards AML showed that the dominant clone at AML stage did not come from blasts identified at the MDS stage but from a subclone only detectable in stem cells at the pre-MDS or MDS stage [48]. At the time of targeted therapies, this notion is of great interest and also explains certain disillusions. Indeed, molecular biology analysis on total population, even with a great depth of reading, will not be able to identify the population really responsible for the leukemic transformation, dooming to failure the attempt to block the leukemic evolution by a targeted therapy based on these global data. Only the early identification of subclones of stem cells particularly apt to develop (mutations known to be of unfavorable prognosis, expression of chemotherapy-resistance genes, etc.) will be able to effectively guide the therapeutic strategy. The single cell mass spectrometry study of Levine et al. [49] consisted in the analysis of 31 proteins under 18 cell stimulation conditions on 15 million cells from 31 AML patients. While in hematopoietic cells from healthy donors the classical surface markers of stem cells correlate with an activation profile, this is not the case in AML samples. Indeed, when the surface phenotype is confronted with the expression and activation profile of functionally and clinically relevant pathways in AMLs, it appears that the profiles are often discordant, leading to the definition of Surface-Defined Primitive Cells (SDPCs) versus Inferred Functionally Primitive Cells (IFPCs). Interestingly, the IFPCs profile has been shown to have a better predictive value for patient survival than the SDPCs profile.

### Therapeutic responses to drugs and targeting

Gilteritinib is a FLT3 inhibitor, effective but not curative in AMLs. The possible emergence of new mutations in relapsed patients has been shown in the total population. McMahon et al. [50] analyzed by a single cell DNA sequencing technique the samples of 4 patients with relapsed AML with RAS mutation. This analysis showed that in all 4 cases the RAS mutation occurred in the same clonal population that had the FLT3 mutation, suggesting that it is the RAS mutation that confers resistance to gilteritinib. In addition, single-cell analysis showed that out of the three AMLs analyzed at diagnosis and relapse, in two patients a mutated NRAS clone pre-existed but only appeared during treatment for the third patient. In addition to these double mutant FLT3/NRAS clones, another patient had a pre-existing FLT3-WT/NRAS-WT subclone containing the IDH1 and SF3B1 mutations, which expanded under gilteritinib [50]. This small series already shows the interest of the single cell approach, which allows highlighting evolutionary patterns of relapse/resistance very different from patient to patient and showing the presence of subclones appearing under gilteritinib or pre-existing, and requiring another approach than the inhibition of FLT3 to allow their eradication. For another FLT3 inhibitor, the single cell analysis showed the existence of polyclonal populations resistant to this drug in patients who relapsed after treatment with quizartinib, with both on- and of target mutations [51].

The single-cell approach also allows a better understanding of the kinetics and pathophysiology of relapses occurring after allogeneic transplantation. Indeed, the single-cell study brings an unequalled analysis by precisely evaluating the post-allograft chimaerism but also by being able to detect and quantify the different leukemic clones according to their phenotypes/mutations and especially to define the co-occurrences of these mutations, which is only possible indirectly and not very precisely by bulk analyses [52]. By this technique two types of relapse could be identified, one based on the development of a clone with a pre-existing mutation and the other on the emergence of a clonal mutation occurring de novo. Nonetheless, single cell analysis has also demonstrated that resistance to chemotherapy can be caused by a non-genetic mechanism via a dynamic transcriptional adaptation [53], arguing for the use of epigenetic therapies [54].

### Disease surveillance and measurable residual disease

The single cell approach has an interest in monitoring MRD. Pellegrino et al. [55] analyzed the evolution of the mutations detected at diagnosis, in CR and during relapse at each stage. At the CR stage, the patient had only 10 mutated cells (7 DNMT3A and 3 TP53) out of the 4384 genotyped cells, with reappearance of these populations during relapse [55]. Furthermore, the relapse analysis showed a significant expansion of the triple-mutant population H2DI/NRAS/ASXL1, demonstrating that any approach that targets only one of the mutations would be doomed to failure. Ediriwickrema et al. [56] analyzed 14 patients, 10 without relapse and 4 with relapse, with a mean of 8177 cells analyzed per point. The analysis of the CR patients showed the existence of mutated cells in 8 of the 10 patients who will relapse and in 3 of the 4 patients in whom no relapse could be observed, the difference not being significant in this small sample size. On the other hand, the number of mutations co-occurring at diagnosis, i.e. present in the same cell, was 2 in patients who relapsed vs. 1 in patients in whom the CR maintained. The small number of patients tested makes any hypothesis risky, but nevertheless this type of data reinforces the interest of the single cell approach since it is not the quantity of mutations that seems important but rather the type of cell distribution, several mutations in the same cell appearing more deleterious than the same mutations spread over different clones.

### Pathogenic mechanism of leukemia

The single cell approach allows the study of the pre-leukemic stages of stem cells and demonstrates their heterogeneity. Indeed, while in some patients all stem cells present at least one mutation-driver and are therefore pre-leukemic hematopoietic stem cells (p-HSC), in other cases this population of p-HSC is undetectable, and mutations are only found at the leukemic blast stage [57]. As stated by the authors, the observed decreased accessibility at HOX transcription factor motifs might mediate the observed retention of stem cell immunophenotype and thus the self-renewal properties of the p-HSC subpopulation. The generation of phylogenetic trees concerning AMLs with NPM1 mutation showed that this mutation, considered as a rather favorable prognosis, was not present in p-HSC, the latter presenting different driver mutations [58]. Nevertheless, when these different p-HSC clones are transplanted into mice, they develop a dominant mutated NPM1 clone 9 times out of 10, although the basic driver mutations were different both in the cases where the NPM1 clone was minor or dominant in the original AML. These data suggest that the NPM1 mutation can only be a powerful driver mutation if it occurs in cells with increased self-renewal capacities acquired through DNM3TA or TET2 mutations for example. In this context of pre-existing driver mutations, the presence of the NPM1 mutation gives a decisive selective advantage for cells with the association of two or more mutations.

The single cell approach is decisive for the evaluation of tumor heterogeneity. Van Galen et al. [59] defined at least 6 tumor cell types (represented by a significant number of cells) according to their transcriptional signature: HSC-like, progenitor-like, granulocyte/macrophage progenitor (GMP)-like, promonocyte-like, monocyte-like and dendritic cell (cDC)-like. These cell subtypes resemble 6 of the 15 cell types identified in normal bone marrow samples. But heterogeneity is also reflected by the different mutations detected in the subpopulations. Indeed, if compared to solid tumors the number of mutations is low in AMLs, these mutations add a level of heterogeneity by the percentage of affected cells (from 1. 8 to 52%) but on the other hand the associations between them of these mutations are at fairly stable levels from leukemia to leukemia: 1 single mutation in 88 to 98% of the cells, 2 mutations in 1.6 to 12% of the cells, 3 mutations between 0 and 0.29% of the cells, and practically no cells with 4 mutations (only 1 cell out of 8200 mutant cells in only one of the 4 AMLs analyzed) [60]. Moreover, this notion may not be extensible to all types of AMLs, with the co-occurrence of receptor-type tyrosine-protein phosphatase T (PTPRT), Cullin Associated And Neddylation Dissociated 1(CAND1) and dedicator of cytokinesis 6 (DOCK6) mutations at least in one case of AML with inv.(16) [61]. Cell-by-cell analysis via pre-sorting allows the frequency of heterozygous versus homozygous mutations to be determined with greater precision than extrapolations from bulk data [62], results that could be more easily obtained with the new techniques of single cell analysis in total population. Finally, single-cell genomics have recently improved our understanding of epistasis among high-frequency mutation [63]. In myeloid neoplasms, mutations in RNA splicing factors most often (> 99% of cases) occur in a mutually exclusive manner. In less than 1% of patients 2 or more splicing factor mutations are detected. The common hypothesis to explain this phenomenon is that the concurrent mutation of 2 or more RNA splicing factors is lethal for the cell. As a consequence, the very rare co-occurrence of RNA splicing factors should involve different subclones. Nonetheless, single-cell analysis has revealed that this co-occurrence is observed in half cases in the same individual cell, but usually with rare amino acid substitutions with reduced effect on RNA splicing, thus explaining the possibility of coexistence in the same cell. Of note, the single cell analysis is not limited to leukemic cells, but can also investigate bone marrow stroma and its interaction with leukemia. In a mouse model, Bariyawno et al. identified 17 different stromal subsets, a regulatory network that was perturbed by the emergence of AML blasts with negative effects on normal stromal cells thus favoring leukemic hematopoiesis [64]. In line with the increased frequency of AML in older patient, the study of Adelman et al. demonstrated that epigenetic reprogramming of aged HSC targets pathways that are also altered in AML [65], probably linked to an increased epigenetic heterogeneity [66].

## Conclusions

**Fig. 1:**
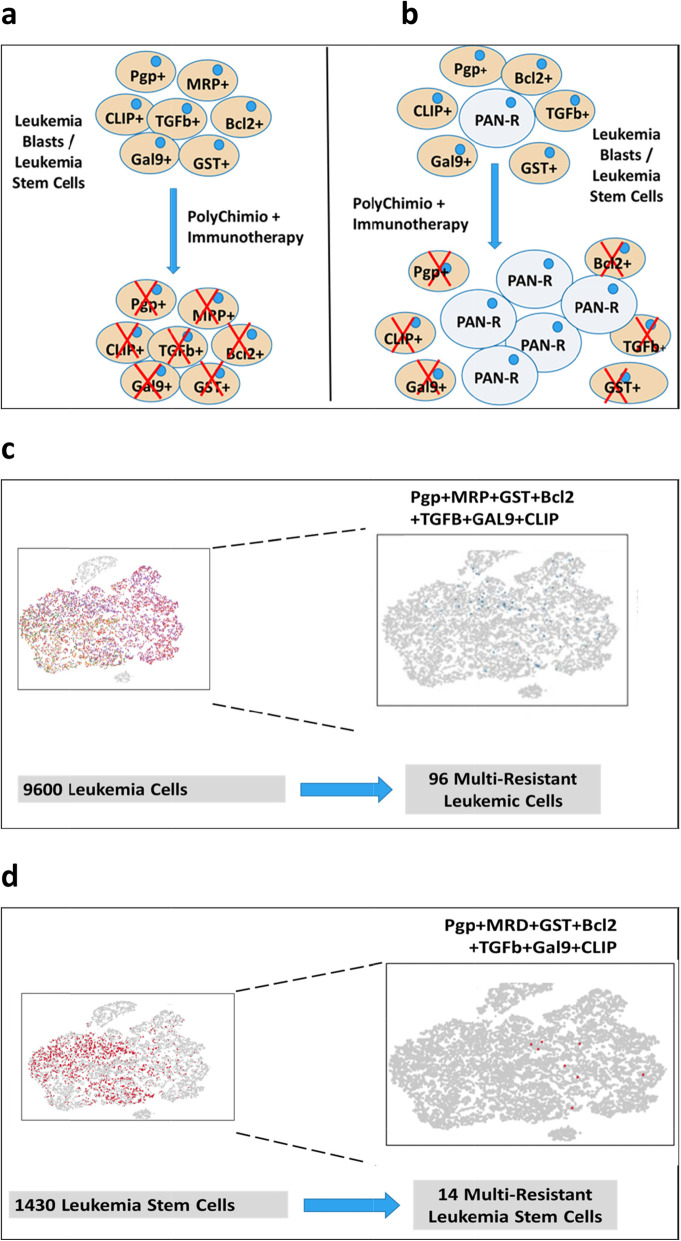
**A:** leukemic cells each bearing a single drug resistance mechanism are depleted by polychemotheray, while **B:** leukemic cells with co-occurrence of various drug resistance mechanisms (Pgp+/MRP+/CLIP+/Gal9+/TGFb+/Bcl2+/GST+ = PAN-Resistant)  hardly are eradicated by polychemotherapy, this co-occurrence in this AML sample is observed in **C:** 1% of leukemia blasts and **D:** 1% of leukemia stem cells defined as CD34 + CD38-CD123+

**Fig. 2:**
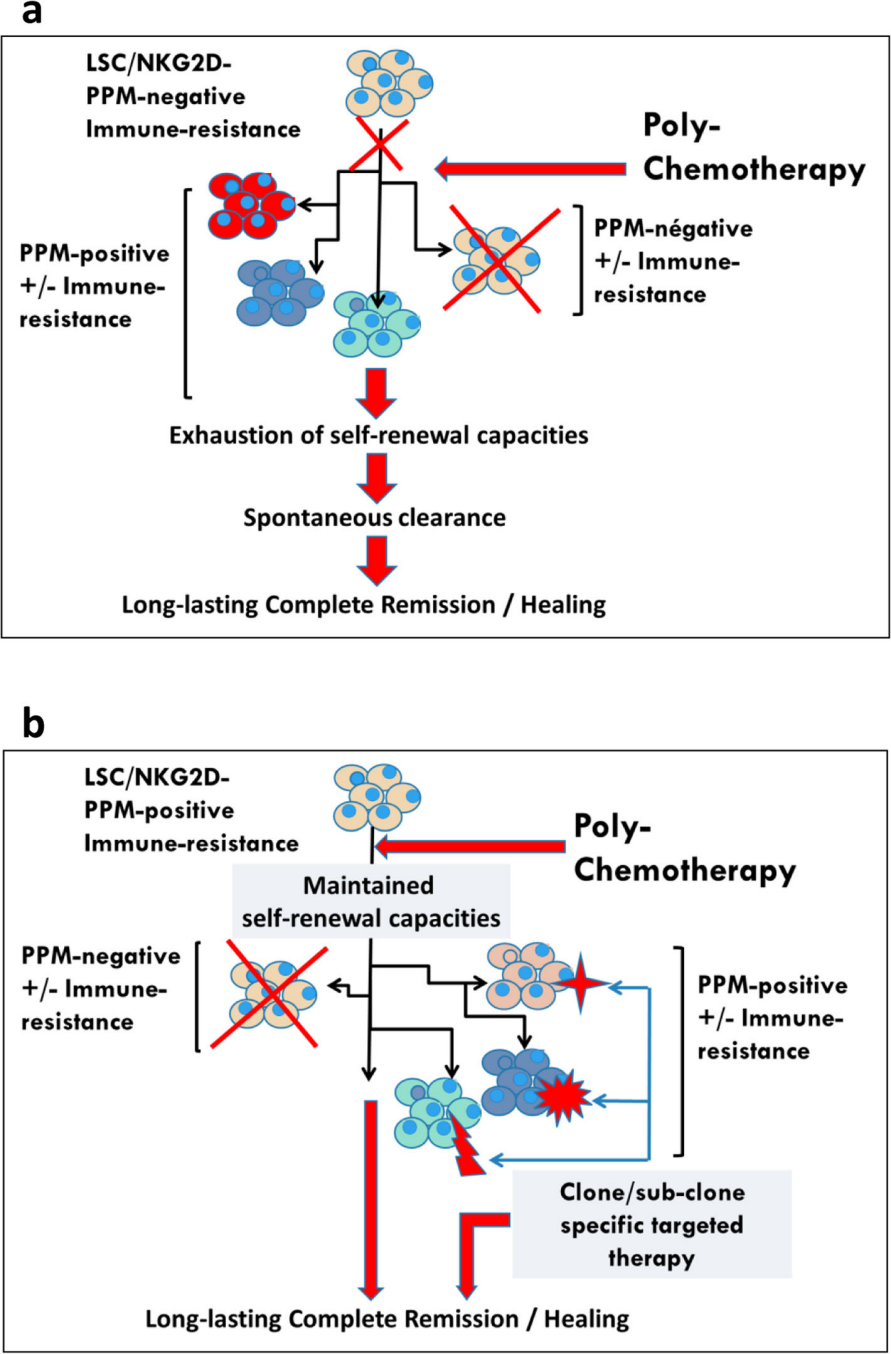
**A:** when no Poor Prognosis Markers (PPM) are detected, classical poly-chemotherapy may attain CR and possibly definitive AML cure, **B:** even in the presence of PPM, if these PPM are evenly distributed between AML blasts, conventional chemotherapy + targeted therapy against residual AML cells may allow leukemia cure

**Fig. 3:**
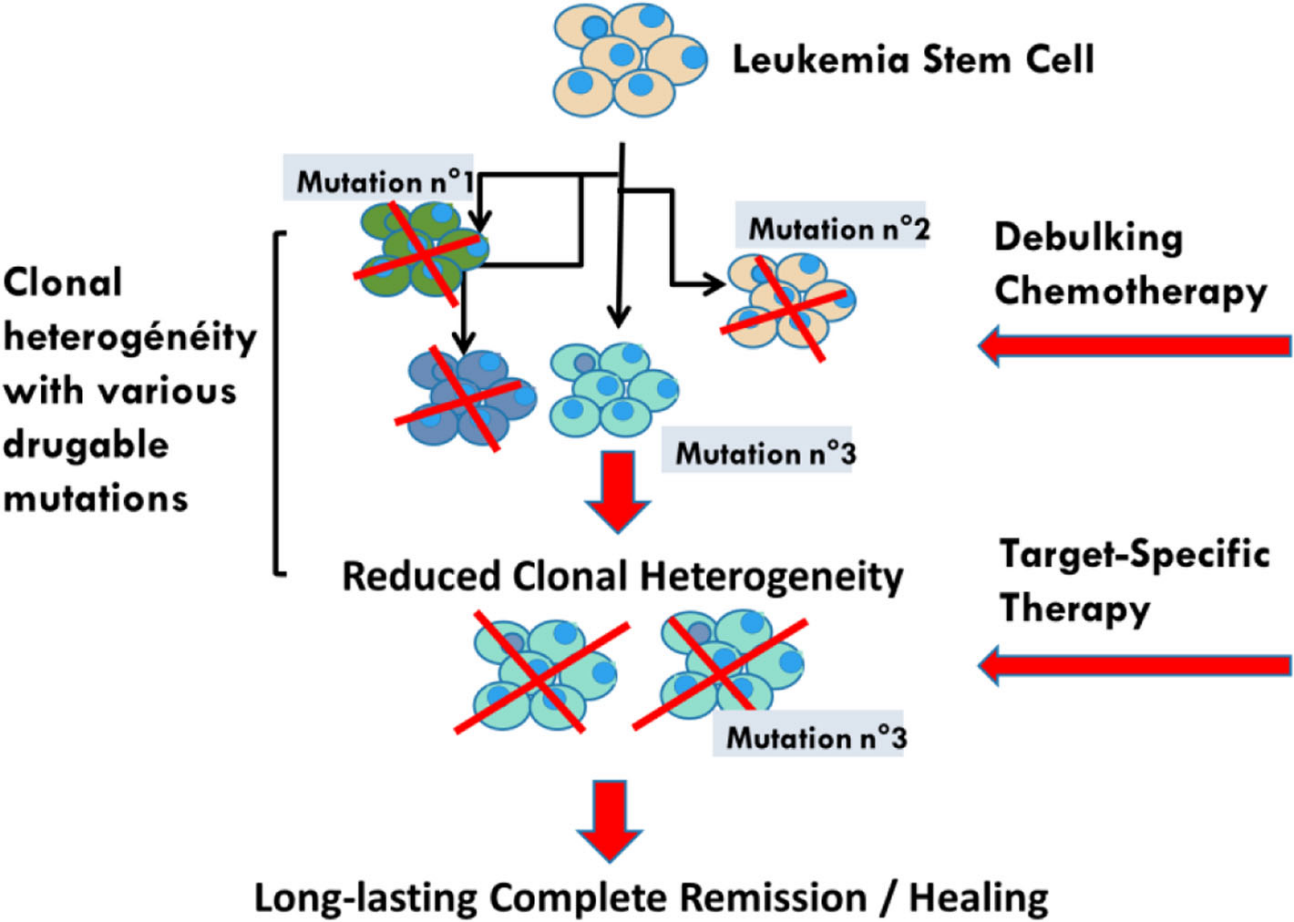
Even if AML cells share many different mutations, classical chemotherapy will have a debulking function associated with clonal heterogeneity reduction, thus increasing the efficiency of specifically targeted therapy

**Fig. 4:**
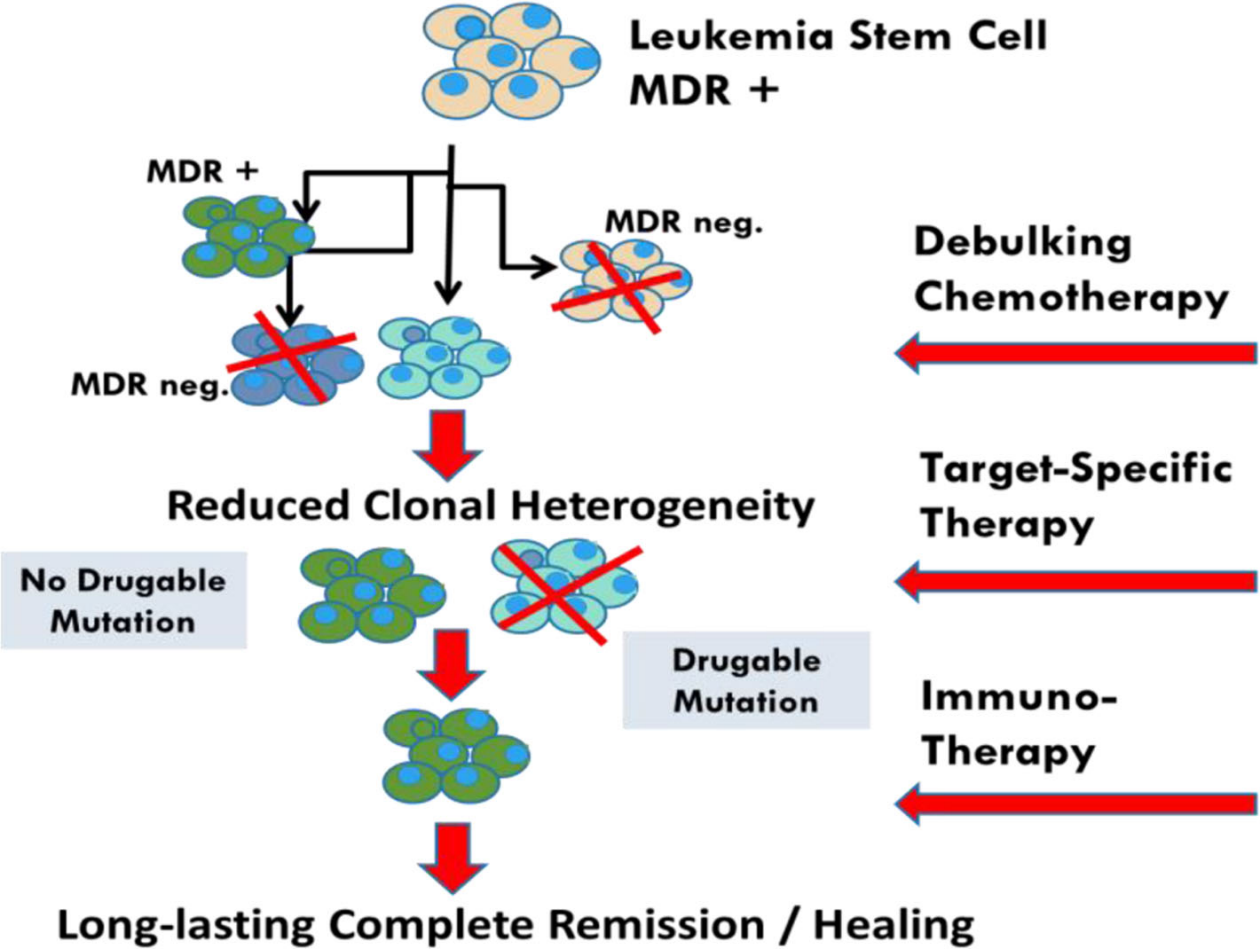
When co-occurrence of multidrug resistance mechanisms at LSC level, classical chemotherapy (+/− sensitizing drugs) will remain useful with the debulking and clonal heterogeneity reduction effects, still allowing the use of targeted therapy followed by passive or active immunotherapy

The progress made in the biological analysis of AMLs has been impressive in recent years, with the virtual abandonment of the FAB morphological classification in favor of the molecular identity card of the European Leukemia Network (ELN) [67]. This risk classification is in constant evolution following new data from NGS/single cell approaches [68]. This molecular classification highlights the numerous mutations with prognostic value but also and perhaps above all those that are drugable, such as the FLT3 mutations as well as IDH1 and IDH2. Compared to solid tumors, the number of mutations found in a given AML is much lower. These two notions have led to the belief that targeted therapies are the ultimate weapon in the treatment of AMLs. Nevertheless, there are many objections to this concept, notably the observations resulting from the various single cell experiments that allow the study of transcribed RNA but also of the DNA sequences themselves on a cell-by-cell basis. There are many lessons to be learned from these techniques, all pointing to the extreme heterogeneity of leukemic blasts. This heterogeneity could be described as spatio-temporal. Spatial heterogeneity, in the sense of t-distributed stochastic neighbor embedding (t-SNE) analysis, is an approach that reduces the dimensionality of blast cells high-dimensional data and enables the data to be visualized in two-dimensional space. It is easy to see by this type of visualization, which groups the cells by the similarity of their gene expression profiles, that within a blast population considered homogeneous by the flow cytometry there are in fact many sub-populations. Indeed, this type of representation rarely shows very narrow population spots. Moreover, the analysis of the expression of certain genes, for example resistance to chemotherapy (L Madaci/B Loriod personal data), shows a significant dispersion at the level of the blast population. The very notion of LSC, defined on the CD34 + CD38- phenotype (with all the variants of phenotypic definition) does not resist the single cell analysis which *in fine* defines various distinct populations having in common the essential characteristic of LSCs, namely the capacities of self-renewal. As for the mutations detected by bulk techniques, single cell analysis shows that they are not present in all tumor cells but only in sub-populations of variable size. Moreover, some drugable mutations may not be present at the LSC population level. The notion of spatial heterogeneity should be complemented by the notion of temporal heterogeneity. Indeed if compared to solid tumors, AMLs present a much smaller number of mutations, it should be noted that leukemias often have a much higher rate of proliferation. In fact, the distribution of sub-populations and mutations is therefore likely to vary very rapidly, either spontaneously, under the pressure of the patient immune response, or under the pressure of conventional chemotherapy drugs or targeted therapies. From these observations we conclude that single cell analysis is a pivotal part of risk stratification in AML. Consequently, it is difficult to develop a therapeutic strategy that would not take into account the initial heterogeneity of AMLs, nor the possibilities of clonal drift over time, i.e. the spatio-temporal heterogeneity of AML blasts and LSCs. This is evidenced by the clinical response that is certainly effective but generally short-lived when targeted therapies are used as monotherapy. The linear evolutionary trajectories model consists in the replacement of a clone-present by a clone derived from itself, when a new mutation or epigenetic modifications occur, giving it a selective advantage through a higher proliferation rate or a greater resistance to the immune response and/or chemotherapies. While this type of evolution may exist, the branched evolutionary model gives rise to divergent clonal populations from the same parent clone. These populations will also eventually compete with each other, leading to the disappearance of one of the branches or possibly to a situation of equilibrium. Whatever the therapeutic approach, it is also necessary to take into account the expected toxicity on progenitors and normal stem cells, necessary for post-chemotherapy hematological reconstitution. What are the steps that should guide our reflection? We have several therapeutic classes at our disposal, such as conventional chemotherapy drugs, demethylating agents, inhibitors of metabolic pathways, targeted therapies and immunotherapy. How can we try to make the most of this armamentarium, impressive at least on paper but much less in practice given the median survival rates? We postulate as a working hypothesis that it is necessary to know individually, i.e. cell by cell, the potential for proliferation, self-renewal and resistance to the different treatments of leukemic cells, in addition to the identification of blasts populations with a drugable mutation. To illustrate the heterogeneity of leukemia at diagnosis regarding the effect of treatment, Fig. 1 (L Madaci/ B Loriod personal data) shows the expression in an AML of seven drug-resistance genes (P-gp, MRP, GSt, Bcl-2, TGFb, Gal-9 and CLIP, for review, [69]). The co-expression of these seven drug resistance markers cited above can be detected in a small percentage of leukemic blasts (Fig. 1, panel A and B) and even in a very discrete but nonetheless significant percentage of LSCs (Fig. 1, panel C and D). This co-expression could explain the relapses, unless we are able to develop a therapeutic pathway to overcome this pan-resistance phenotype. It is also mandatory to repeat this analysis after each therapeutic assault to monitor leukemia heterogeneity evolution due to the clonal drift accelerated by the pressure exerted by chemo/immunotherapy. The co-expression of seven drug-related resistance genes in a significant number of LSC underlines the pivotal role of a specific analysis at diagnosis and during treatment of this subpopulation. In a very simplistic model, if the expression of treatment-resistance genes (even co-expression) is detected in the more mature blasts population and not in LSC, this leukemia may be considered of good prognosis since the LSC will be eliminated by the directly by the chemo-immunotherapy (Fig. 2a) or by the additional use of targeted monotherapy or doublets/triplets to cover all detected mutations (Fig. 2b). Single-cell analysis will also make it possible to adapt the most useful sequences during treatment according to the fluctuations observed in the blast populations of the residual disease. Starting treatment with conventional chemotherapy will reduce tumor heterogeneity before using specific targeted therapies (Fig. 3). In the difficult case where one or more mechanisms of resistance to conventional drugs exist in the LSCs (Fig. 4), the addition of sensitizing drugs or drugs with specific effect on LSC [70] will be used when possible, but this is not always the case (overexpression of MRP1, too ubiquitous to be targeted). In this case, classical chemotherapy will have a debulking role, reducing tumor heterogeneity and then, in some patients, allowing the use of targeted therapies. Finally, single cell analysis of whole peripheral blood/bone marrow involves not only leukemic cells but also part of its immunological environment, i.e. T lymphocytes, NK cells [71] and even dendritic cell populations. These data could possibly improve the positioning of the different immunotherapy approaches, i.e. monoclonal antibodies, cytokines, allogenic transplantation or T-CAR cells.

## Data Availability

The datasets used and/or analysed during the current study are available from the corresponding author on reasonable request.

## Abbreviations

ALL: Acute lymphoblastic leukemia
AML: Acute myeloid leukemia
IDH1/2: Isocitrate deshydrogenase1/2
ASXL1: Additional SeX combs Like 1
BAX: Bcl-2-associated X protein
BCL2: B-cell lymphoma 2
BTK: Bruton tyrosine kinase
CAND1: Cullin associated and neddylation dissociated 1
CEBPA: CCAAT/enhancer-binding protein alpha
CITE-seq: Cellular Indexing of transcriptomic and epitopes by sequencing
CLL: Chronic lymphocytic leukemia
CR: Complete remission
Cri: CR with incomplete hematologic recovery
CYP3: Cytochrome P450
DC: Dendritic cells
DNMT3: DNA-MethylTransferase 3
DOCK6: Dedicator of cytokinesis 6
EFS: Event-free survival
FGF: Fibroblast growth factor
ELN: European leukemia network
FGFR: FGF receptor
FLT3: Fms-like tyrosine kinase 3
GLI1: Glioma-associated oncogene homolog
GMP: Granulocyte/macrophage progenitor
Hh: Hedgehog
HSC: Human normal hematopoietic stem cell
IFPCs: Inferred functionally primitive cells
ITD: Internal tandem duplication
IL6ST: Interleukin 6 signal transducer
JMJD2A: Jumonji C domaine-containing demethylase
KG: Ketoglutatrate
HG: Hyrdoxyglutarate
LIFR: Leukemia inhibitory factor receptor
LSC: Leukemia stem cell
MDS: Myelodysplastic syndrome
MOMP: Mitochondrial outer membrane permeabilization
MRD: Measurable residual disease
NGS: Next generation sequencing
NK: Natural killer
NOXA: Latin for damage
NPM1: Nucleophosmin
NRAS: Neuroblastoma rat sarcoma
ORR: overall response rate
OS: Overall survival
PDGFR: Platelet derived growth factor receptor
PTPN11: Tyrosine-protein phosphatase non-receptor type 11
PTRPT: Receptor-type tyrosine-protein phosphatase T
R-IPSS: revised-international prognostic scoring system
RNA: Ribonucleic acid
DNA: Desoxyribonucleic acid
ROS: Reactive oxygen species
RUNX1: Runt-related transcription factor 1
SDPCs: Surface-defined primitive cells
SRSF2: Serine/arginine-rich splicing factor 2
T-CAR cells: Chimeric antigen receptors T lymphocytes
TET2: Ten-eleven translocation 2
TGF: Tumor growth factor
VEGFR: Vascular endothelial growth factor receptor
WT1: Wilm’s tumor 1

## References

[1] McCombie WR, McPherson JD, Mardis ER. Next-generation sequencing technologies. Cold Spring Harb Perspect Med. 2019;9(11). 10.1101/cshperspect.a036798. 10.1101/cshperspect.a036798 PMC6824406 30478097

[2] Rozenblatt-Rosen O, Regev A, Oberdoerffer P, Nawy T, Hupalowska A, Rood JE, Ashenberg O, Cerami E, Coffey RJ, Demir E, Ding L, Esplin ED, Ford JM, Goecks J, Ghosh S, Gray JW, Guinney J, Hanlon SE, Hughes SK, Hwang ES, Iacobuzio-Donahue CA, Jané-Valbuena J, Johnson BE, Lau KS, Lively T, Mazzilli SA, Pe’er D, Santagata S, Shalek AK, Schapiro D, Snyder MP, Sorger PK, Spira AE, Srivastava S, Tan K, West RB, Williams EH, Aberle D, Achilefu SI, Ademuyiwa FO, Adey AC, Aft RL, Agarwal R, Aguilar RA, Alikarami F, Allaj V, Amos C, Anders RA, Angelo MR, Anton K, Ashenberg O, Aster JC, Babur O, Bahmani A, Balsubramani A, Barrett D, Beane J, Bender DE, Bernt K, Berry L, Betts CB, Bletz J, Blise K, Boire A, Boland G, Borowsky A, Bosse K, Bott M, Boyden E, Brooks J, Bueno R, Burlingame EA, Cai Q, Campbell J, Caravan W, Cerami E, Chaib H, Chan JM, Chang YH, Chatterjee D, Chaudhary O, Chen AA, Chen B, Chen C, Chen CH, Chen F, Chen YA, Chheda MG, Chin K, Chiu R, Chu SK, Chuaqui R, Chun J, Cisneros L, Coffey RJ, Colditz GA, Cole K, Collins N, Contrepois K, Coussens LM, Creason AL, Crichton D, Curtis C, Davidsen T, Davies SR, de Bruijn I, Dellostritto L, de Marzo A, Demir E, DeNardo DG, Diep D, Ding L, Diskin S, Doan X, Drewes J, Dubinett S, Dyer M, Egger J, Eng J, Engelhardt B, Erwin G, Esplin ED, Esserman L, Felmeister A, Feiler HS, Fields RC, Fisher S, Flaherty K, Flournoy J, Ford JM, Fortunato A, Frangieh A, Frye JL, Fulton RS, Galipeau D, Gan S, Gao J, Gao L, Gao P, Gao VR, Geiger T, George A, Getz G, Ghosh S, Giannakis M, Gibbs DL, Gillanders WE, Goecks J, Goedegebuure SP, Gould A, Gowers K, Gray JW, Greenleaf W, Gresham J, Guerriero JL, Guha TK, Guimaraes AR, Guinney J, Gutman D, Hacohen N, Hanlon S, Hansen CR, Harismendy O, Harris KA, Hata A, Hayashi A, Heiser C, Helvie K, Herndon JM, Hirst G, Hodi F, Hollmann T, Horning A, Hsieh JJ, Hughes S, Huh WJ, Hunger S, Hwang SE, Iacobuzio-Donahue CA, Ijaz H, Izar B, Jacobson CA, Janes S, Jané-Valbuena J, Jayasinghe RG, Jiang L, Johnson BE, Johnson B, Ju T, Kadara H, Kaestner K, Kagan J, Kalinke L, Keith R, Khan A, Kibbe W, Kim AH, Kim E, Kim J, Kolodzie A, Kopytra M, Kotler E, Krueger R, Krysan K, Kundaje A, Ladabaum U, Lake BB, Lam H, Laquindanum R, Lau KS, Laughney AM, Lee H, Lenburg M, Leonard C, Leshchiner I, Levy R, Li J, Lian CG, Lim KH, Lin JR, Lin Y, Liu Q, Liu R, Lively T, Longabaugh WJR, Longacre T, Ma CX, Macedonia MC, Madison T, Maher CA, Maitra A, Makinen N, Makowski D, Maley C, Maliga Z, Mallo D, Maris J, Markham N, Marks J, Martinez D, Mashl RJ, Masilionais I, Mason J, Massagué J, Massion P, Mattar M, Mazurchuk R, Mazutis L, Mazzilli SA, McKinley ET, McMichael JF, Merrick D, Meyerson M, Miessner JR, Mills GB, Mills M, Mondal SB, Mori M, Mori Y, Moses E, Mosse Y, Muhlich JL, Murphy GF, Navin NE, Nawy T, Nederlof M, Ness R, Nevins S, Nikolov M, Nirmal AJ, Nolan G, Novikov E, Oberdoerffer P, O’Connell B, Offin M, Oh ST, Olson A, Ooms A, Ossandon M, Owzar K, Parmar S, Patel T, Patti GJ, Pe’er D, Pe'er I, Peng T, Persson D, Petty M, Pfister H, Polyak K, Pourfarhangi K, Puram SV, Qiu Q, Quintanal-Villalonga Á, Raj A, Ramirez-Solano M, Rashid R, Reeb AN, Regev A, Reid M, Resnick A, Reynolds SM, Riesterer JL, Rodig S, Roland JT, Rosenfield S, Rotem A, Roy S, Rozenblatt-Rosen O, Rudin CM, Ryser MD, Santagata S, Santi-Vicini M, Sato K, Schapiro D, Schrag D, Schultz N, Sears CL, Sears RC, Sen S, Sen T, Shalek A, Sheng J, Sheng Q, Shoghi KI, Shrubsole MJ, Shyr Y, Sibley AB, Siex K, Simmons AJ, Singer DS, Sivagnanam S, Slyper M, Snyder MP, Sokolov A, Song SK, Sorger PK, Southard-Smith A, Spira A, Srivastava S, Stein J, Storm P, Stover E, Strand SH, Su T, Sudar D, Sullivan R, Surrey L, Suvà M, Tan K, Terekhanova NV, Ternes L, Thammavong L, Thibault G, Thomas GV, Thorsson V, Todres E, Tran L, Tyler M, Uzun Y, Vachani A, van Allen E, Vandekar S, Veis DJ, Vigneau S, Vossough A, Waanders A, Wagle N, Wang LB, Wendl MC, West R, Williams EH, Wu CY, Wu H, Wu HY, Wyczalkowski MA, Xie Y, Yang X, Yapp C, Yu W, Yuan Y, Zhang D, Zhang K, Zhang M, Zhang N, Zhang Y, Zhao Y, Zhou DC, Zhou Z, Zhu H, Zhu Q, Zhu X, Zhu Y, Zhuang X (2020). The human tumor atlas network: charting tumor transitions across space and time at single-cell resolution. Cell.

[3] Stuart T, Satija R (2019). Integrative single-cell analysis. Nat Rev Genet.

[4] McKinnon KM (2018). Flow cytometry: an overview. Curr Protoc Immunol.

[5] Han L, Qiu P, Zeng Z, Jorgensen JL, Mak DH, Burks JK, Schober W, McQueen TJ, Cortes J, Tanner SD, Roboz GJ, Kantarjian HM, Kornblau SM, Guzman ML, Andreeff M, Konopleva M (2015). Single-cell mass cytometry reveals intracellular survival/proliferative signaling in FLT3-ITD-mutated AML stem/progenitor cells. Cytometry A.

[6] Zeng Z, Konopleva M, Andreeff M (1633). Single-cell mass cytometry of acute myeloid leukemia and leukemia stem/progenitor cells. Methods Mol Biol.

[7] Stoeckius M, Zheng S, Houck-Loomis B, Hao S, Yeung BZ, Mauck WM, Smibert P, Satija R (2018). Cell hashing with barcoded antibodies enables multiplexing and doublet detection for single cell genomics. Genome Biol.

[8] Stoeckius M, Hafemeister C, Stephenson W, Houck-Loomis B, Chattopadhyay PK, Swerdlow H, Satija R, Smibert P (2017). Simultaneous epitope and transcriptome measurement in single cells. Nat Methods.

[9] Papaemmanuil E, Gerstung M, Bullinger L, Gaidzik VI, Paschka P, Roberts ND, Potter NE, Heuser M, Thol F, Bolli N, Gundem G, van Loo P, Martincorena I, Ganly P, Mudie L, McLaren S, O’Meara S, Raine K, Jones DR, Teague JW, Butler AP, Greaves MF, Ganser A, Döhner K, Schlenk RF, Döhner H, Campbell PJ (2016). Genomic classification and prognosis in acute myeloid leukemia. N Engl J Med.

[10] DiNardo CD, Stein EM, de BS, Roboz GJ, Altman JK, Mims AS, et al. (2018). Durable remissions with Ivosidenib in IDH1-mutated relapsed or refractory AML. N Engl J Med.

[11] DiNardo CD (2018). Ivosidenib in IDH1-mutated acute myeloid leukemia. N Engl J Med.

[12] Kurt H, Bueso-Ramos CE, Khoury JD, Routbort MJ, Kanagal-Shamanna R, Patel UV, Jorgensen JL, Wang SA, Ravandi F, DiNardo C, Luthra R, Medeiros LJ, Patel KP (2018). Characterization of IDH1 p.R132H mutant clones using mutation-specific antibody in myeloid neoplasms. Am J Surg Pathol.

[13] Li Y, Connarn JN, Chen J, Tong Z, Palmisano M, Zhou S (2019). Modeling and simulation of the endogenous CYP3A induction marker 4beta-hydroxycholesterol during enasidenib treatment. Clin Pharmacol.

[14] Coutts RT, Su P, Baker GB (1994). Involvement of CYP2D6, CYP3A4, and other cytochrome P-450 isozymes in N-dealkylation reactions. J Pharmacol Toxicol Methods.

[15] Tong Z, Atsriku C, Yerramilli U, Wang X, Li Y, Reyes J, Fan B, Yang H, Hoffmann M, Surapaneni S (2019). Absorption, distribution, metabolism and excretion of an isocitrate dehydrogenase-2 inhibitor enasidenib in rats and humans. Xenobiotica.

[16] Quek L, David MD, Kennedy A, Metzner M, Amatangelo M, Shih A, Stoilova B, Quivoron C, Heiblig M, Willekens C, Saada V, Alsafadi S, Vijayabaskar MS, Peniket A, Bernard OA, Agresta S, Yen K, MacBeth K, Stein E, Vassiliou GS, Levine R, de Botton S, Thakurta A, Penard-Lacronique V, Vyas P (2018). Clonal heterogeneity of acute myeloid leukemia treated with the IDH2 inhibitor enasidenib. Nat Med.

[17] Intlekofer AM, Shih AH, Wang B, Nazir A, Rustenburg AS, Albanese SK, Patel M, Famulare C, Correa FM, Takemoto N, Durani V, Liu H, Taylor J, Farnoud N, Papaemmanuil E, Cross JR, Tallman MS, Arcila ME, Roshal M, Petsko GA, Wu B, Choe S, Konteatis ZD, Biller SA, Chodera JD, Thompson CB, Levine RL, Stein EM (2018). Acquired resistance to IDH inhibition through trans or cis dimer-interface mutations. Nature.

[18] Long B, Wang LX, Zheng FM, Lai SP, Xu DR, Hu Y, Lin DJ, Zhang XZ, Dong L, Long ZJ, Tong XZ, Liu Q (2016). Targeting GLI1 suppresses cell growth and enhances Chemosensitivity in CD34+ enriched acute myeloid leukemia progenitor cells. Cell Physiol Biochem.

[19] Costello RT, Mallet F, Gaugler B, Sainty D, Arnoulet C, Gastaut JA (2000). Human acute myeloid leukemia CD34+/CD38- progenitor cells have decreased sensitivity to chemotherapy and Fas-induced apoptosis, reduced immunogenicity, and impaired dendritic cell transformation capacities. Cancer Res.

[20] Martinelli G, Oehler VG, Papayannidis C, Courtney R, Shaik MN, Zhang X, O'Connell A, McLachlan KR, Zheng X, Radich J, Baccarani M, Kantarjian HM, Levin WJ, Cortes JE, Jamieson C (2015). Treatment with PF-04449913, an oral smoothened antagonist, in patients with myeloid malignancies: a phase 1 safety and pharmacokinetics study. Lancet Haematol.

[21] Cortes JE, Heidel FH, Hellmann A, Fiedler W, Smith BD, Robak T, Montesinos P, Pollyea DA, DesJardins P, Ottmann O, Ma WW, Shaik MN, Laird AD, Zeremski M, O’Connell A, Chan G, Heuser M (2019). Randomized comparison of low dose cytarabine with or without glasdegib in patients with newly diagnosed acute myeloid leukemia or high-risk myelodysplastic syndrome. Leukemia.

[22] Tremblay G, Westley T, Cappelleri JC, Arondekar B, Chan G, Bell TJ, Briggs A (2019). Overall survival of glasdegib in combination with low-dose cytarabine, azacitidine, and decitabine among adult patients with previously untreated AML: comparative effectiveness using simulated treatment comparisons. Clinicoecon Outcomes Res.

[23] Cortes JE, Douglas SB, Wang ES, Merchant A, Oehler VG, Arellano M (2018). Glasdegib in combination with cytarabine and daunorubicin in patients with AML or high-risk MDS: phase 2 study results. Am J Hematol.

[24] Sallman DA, Komrokji RS, Sweet KL, Mo Q, McGraw KL, Duong VH (2019). A phase 2 trial of the oral smoothened inhibitor glasdegib in refractory myelodysplastic syndromes (MDS). Leuk Res.

[25] Smith CC (2019). The growing landscape of FLT3 inhibition in AML. Hematol Am Soc Hematol Educ Program.

[26] Stone RM, Mandrekar SJ, Sanford BL, Laumann K, Geyer S, Bloomfield CD, Thiede C, Prior TW, Döhner K, Marcucci G, Lo-Coco F, Klisovic RB, Wei A, Sierra J, Sanz MA, Brandwein JM, de Witte T, Niederwieser D, Appelbaum FR, Medeiros BC, Tallman MS, Krauter J, Schlenk RF, Ganser A, Serve H, Ehninger G, Amadori S, Larson RA, Döhner H (2017). Midostaurin plus chemotherapy for acute myeloid leukemia with a FLT3 mutation. N Engl J Med.

[27] Lim Y, Gondek L, Li L, Wang Q, Ma H, Chang E (2015). Integration of Hedgehog and mutant FLT3 signaling in myeloid leukemia. Sci Transl Med.

[28] Perl AE, Martinelli G, Cortes JE, Neubauer A, Berman E, Paolini S, Montesinos P, Baer MR, Larson RA, Ustun C, Fabbiano F, Erba HP, di Stasi A, Stuart R, Olin R, Kasner M, Ciceri F, Chou WC, Podoltsev N, Recher C, Yokoyama H, Hosono N, Yoon SS, Lee JH, Pardee T, Fathi AT, Liu C, Hasabou N, Liu X, Bahceci E, Levis MJ (2019). Gilteritinib or chemotherapy for relapsed or refractory FLT3-mutated AML. N Engl J Med.

[29] Antar AI, Otrock ZK, Jabbour E, Mohty M, Bazarbachi A (2020). FLT3 inhibitors in acute myeloid leukemia: ten frequently asked questions. Leukemia.

[30] Lauria F, Raspadori D, Rondelli D, Ventura MA, Fiacchini M, Visani G, Forconi F, Tura S (1997). High bcl-2 expression in acute myeloid leukemia cells correlates with CD34 positivity and complete remission rate. Leukemia.

[31] Konopleva M, Pollyea DA, Potluri J, Chyla B, Hogdal L, Busman T, McKeegan E, Salem AH, Zhu M, Ricker JL, Blum W, DiNardo CD, Kadia T, Dunbar M, Kirby R, Falotico N, Leverson J, Humerickhouse R, Mabry M, Stone R, Kantarjian H, Letai A (2016). Efficacy and biological correlates of response in a phase II study of Venetoclax monotherapy in patients with acute myelogenous leukemia. Cancer Discov.

[32] Chyla B, Daver N, Doyle K, McKeegan E, Huang X, Ruvolo V (2018). Genetic biomarkers of sensitivity and resistance to Venetoclax monotherapy in patients with relapsed acute myeloid leukemia. Am J Hematol.

[33] Wei AH, Montesinos P, Ivanov V, DiNardo CD, Novak J, Laribi K (2020). Venetoclax plus LDAC for patients with untreated AML ineligible for intensive chemotherapy: phase 3 randomized placebo-controlled trial. Blood.

[34] DiNardo CD, Pratz K, Pullarkat V, Jonas BA, Arellano M, Becker PS (2019). Venetoclax combined with decitabine or azacitidine in treatment-naive, elderly patients with acute myeloid leukemia. Blood.

[35] Gutman JA, Pollyea DA (2020). Hypomethylating agents with venetoclax: have we discovered the holy grail?. Curr Opin Hematol.

[36] Jin S, Cojocari D, Purkal JJ, Popovic R, Talaty NN, Xiao Y, Solomon LR, Boghaert ER, Leverson JD, Phillips DC (2020). 5-Azacitidine induces NOXA to prime AML cells for Venetoclax-mediated apoptosis. Clin Cancer Res.

[37] DiNardo CD, Tiong IS, Quaglieri A, MacRaild S, Loghavi S, Brown FC (2020). Molecular patterns of response and treatment failure after frontline venetoclax combinations in older patients with AML. Blood.

[38] Pei S, Pollyea DA, Gustafson A, Stevens BM, Minhajuddin M, Fu R, Riemondy KA, Gillen AE, Sheridan RM, Kim J, Costello JC, Amaya ML, Inguva A, Winters A, Ye H, Krug A, Jones CL, Adane B, Khan N, Ponder J, Schowinsky J, Abbott D, Hammes A, Myers JR, Ashton JM, Nemkov T, D'Alessandro A, Gutman JA, Ramsey HE, Savona MR, Smith CA, Jordan CT (2020). Monocytic subclones confer resistance to Venetoclax-based therapy in patients with acute myeloid leukemia. Cancer Discov.

[39] Brinton LT, Zhang P, Williams K, Canfield D, Orwick S, Sher S, Wasmuth R, Beaver L, Cempre C, Skinner J, Cannon M, Govande M, Harrington B, Lehman A, Byrd JC, Lapalombella R, Blachly JS (2020). Synergistic effect of BCL2 and FLT3 co-inhibition in acute myeloid leukemia. J Hematol Oncol.

[40] Komarova NL, Burger JA, Wodarz D (2014). Evolution of ibrutinib resistance in chronic lymphocytic leukemia (CLL). Proc Natl Acad Sci U S A.

[41] Zhu Y, Huang Y, Tan Y, Zhao W, Tian Q (2020). Single-cell RNA sequencing in hematological diseases. Proteomics.

[42] Gupta SD, Sachs Z (2017). Novel single-cell technologies in acute myeloid leukemia research. Transl Res.

[43] Povinelli BJ, Rodriguez-Meira A, Mead AJ (2018). Single cell analysis of normal and leukemic hematopoiesis. Mol Asp Med.

[44] Won EJ, Kim HR, Park RY, Choi SY, Shin JH, Suh SP, Ryang DW, Szardenings M, Shin MG (2015). Direct confirmation of quiescence of CD34+. BMC Cancer.

[45] Sachs K, Sarver AL, Noble-Orcutt KE, LaRue RS, Antony ML, Chang D (2020). Single-cell gene expression analyses reveal distinct self-renewing and proliferating subsets in the leukemia stem cell compartment in acute myeloid leukemia. Cancer Res.

[46] Zhu H, Zhang L, Wu Y, Dong B, Guo W, Wang M, et al. T-ALL leukemia stem cell 'stemness' is epigenetically controlled by the master regulator SPI1. Elife. 2018;7. 10.7554/eLife.38314. 10.7554/eLife.38314 PMC6251627 30412053

[47] Nazha A (2018). The MDS genomics-prognosis symbiosis. Hematol Am Soc Hematol Educ Program.

[48] Chen J, Kao YR, Sun D, Todorova TI, Reynolds D, Narayanagari SR, Montagna C, Will B, Verma A, Steidl U (2019). Myelodysplastic syndrome progression to acute myeloid leukemia at the stem cell level. Nat Med.

[49] Levine JH, Simonds EF, Bendall SC, Davis KL, Amir e, Tadmor MD, et al. (2015). Data-driven phenotypic dissection of AML reveals progenitor-like cells that correlate with prognosis. Cell.

[50] McMahon CM, Ferng T, Canaani J, Wang ES, Morrissette JJD, Eastburn DJ (2019). Clonal selection with RAS pathway activation mediates secondary clinical resistance to selective FLT3 inhibition in acute myeloid leukemia. Cancer Discov.

[51] Smith CC, Paguirigan A, Jeschke GR, Lin KC, Massi E, Tarver T, Chin CS, Asthana S, Olshen A, Travers KJ, Wang S, Levis MJ, Perl AE, Radich JP, Shah NP (2017). Heterogeneous resistance to quizartinib in acute myeloid leukemia revealed by single-cell analysis. Blood.

[52] Xu L, Durruthy-Durruthy R, Eastburn DJ, Pellegrino M, Shah O, Meyer E, Zehnder J (2019). Clonal evolution and changes in two AML patients detected with a novel single-cell DNA sequencing platform. Sci Rep.

[53] Bell CC, Fennell KA, Chan YC, Rambow F, Yeung MM, Vassiliadis D, Lara L, Yeh P, Martelotto LG, Rogiers A, Kremer BE, Barbash O, Mohammad HP, Johanson TM, Burr ML, Dhar A, Karpinich N, Tian L, Tyler DS, MacPherson L, Shi J, Pinnawala N, Yew Fong C, Papenfuss AT, Grimmond SM, Dawson SJ, Allan RS, Kruger RG, Vakoc CR, Goode DL, Naik SH, Gilan O, Lam EYN, Marine JC, Prinjha RK, Dawson MA (2019). Targeting enhancer switching overcomes non-genetic drug resistance in acute myeloid leukaemia. Nat Commun.

[54] Fennell KA, Bell CC, Dawson MA (2019). Epigenetic therapies in acute myeloid leukemia: where to from here?. Blood.

[55] Pellegrino M, Sciambi A, Treusch S, Durruthy-Durruthy R, Gokhale K, Jacob J, Chen TX, Geis JA, Oldham W, Matthews J, Kantarjian H, Futreal PA, Patel K, Jones KW, Takahashi K, Eastburn DJ (2018). High-throughput single-cell DNA sequencing of acute myeloid leukemia tumors with droplet microfluidics. Genome Res.

[56] Ediriwickrema A, Aleshin A, Reiter JG, Corces MR, Kohnke T, Stafford M (2020). Single-cell mutational profiling enhances the clinical evaluation of AML MRD. Blood Adv.

[57] Corces MR, Buenrostro JD, Wu B, Greenside PG, Chan SM, Koenig JL, Snyder MP, Pritchard JK, Kundaje A, Greenleaf WJ, Majeti R, Chang HY (2016). Lineage-specific and single-cell chromatin accessibility charts human hematopoiesis and leukemia evolution. Nat Genet.

[58] Potter N, Miraki-Moud F, Ermini L, Titley I, Vijayaraghavan G, Papaemmanuil E, Campbell P, Gribben J, Taussig D, Greaves M (2019). Single cell analysis of clonal architecture in acute myeloid leukaemia. Leukemia.

[59] Van GP, Hovestadt V, Wadsworth Ii MH, Hughes TK, Griffin GK, Battaglia S (2019). Single-cell RNA-Seq reveals AML hierarchies relevant to disease progression and immunity. Cell.

[60] Petti AA, Williams SR, Miller CA, Fiddes IT, Srivatsan SN, Chen DY, Fronick CC, Fulton RS, Church DM, Ley TJ (2019). A general approach for detecting expressed mutations in AML cells using single cell RNA-sequencing. Nat Commun.

[61] Niemoller C, Renz N, Bleul S, Blagitko-Dorfs N, Greil C, Yoshida K (2016). Single cell genotyping of exome sequencing-identified mutations to characterize the clonal composition and evolution of inv(16) AML in a CBL mutated clonal hematopoiesis. Leuk Res.

[62] Paguirigan AL, Smith J, Meshinchi S, Carroll M, Maley C, Radich JP (2015). Single-cell genotyping demonstrates complex clonal diversity in acute myeloid leukemia. Sci Transl Med.

[63] Taylor J, Mi X, North K, Binder M, Penson A, Lasho T, Knorr K, Haddadin M, Liu B, Pangallo J, Benbarche S, Wiseman D, Tefferi A, Halene S, Liang Y, Patnaik MM, Bradley RK, Abdel-Wahab O (2020). Single-cell genomics reveals the genetic and molecular bases for escape from mutational epistasis in myeloid neoplasms. Blood.

[64] Baryawno N, Przybylski D, Kowalczyk MS, Kfoury Y, Severe N, Gustafsson K, Kokkaliaris KD, Mercier F, Tabaka M, Hofree M, Dionne D, Papazian A, Lee D, Ashenberg O, Subramanian A, Vaishnav ED, Rozenblatt-Rosen O, Regev A, Scadden DT (2019). A cellular taxonomy of the bone marrow stroma in homeostasis and leukemia. Cell.

[65] Adelman ER, Huang HT, Roisman A, Olsson A, Colaprico A, Qin T, Lindsley RC, Bejar R, Salomonis N, Grimes HL, Figueroa ME (2019). Aging human hematopoietic stem cells manifest profound epigenetic reprogramming of enhancers that may predispose to leukemia. Cancer Discov.

[66] Li S, Garrett-Bakelman FE, Chung SS, Sanders MA, Hricik T, Rapaport F, Patel J, Dillon R, Vijay P, Brown AL, Perl AE, Cannon J, Bullinger L, Luger S, Becker M, Lewis ID, To LB, Delwel R, Löwenberg B, Döhner H, Döhner K, Guzman ML, Hassane DC, Roboz GJ, Grimwade D, Valk PJM, D'Andrea RJ, Carroll M, Park CY, Neuberg D, Levine R, Melnick AM, Mason CE (2016). Distinct evolution and dynamics of epigenetic and genetic heterogeneity in acute myeloid leukemia. Nat Med.

[67] Dohner H, Estey E, Grimwade D, Amadori S, Appelbaum FR, Buchner T (2017). Diagnosis and management of AML in adults: 2017 ELN recommendations from an international expert panel. Blood.

[68] Eisfeld AK, Kohlschmidt J, Mims A, Nicolet D, Walker CJ, Blachly JS, Carroll AJ, Papaioannou D, Kolitz JE, Powell BE, Stone RM, de la Chapelle A, Byrd JC, Mrózek K, Bloomfield CD (2020). Additional gene mutations may refine the 2017 European LeukemiaNet classification in adult patients with de novo acute myeloid leukemia aged <60 years. Leukemia.

[69] Zhang J, Gu Y, Chen B (2019). Mechanisms of drug resistance in acute myeloid leukemia. Onco Targets Ther.

[70] Venton G, Perez-Alea M, Baier C, Fournet G, Quash G, Labiad Y (2016). Aldehyde dehydrogenases inhibition eradicates leukemia stem cells while sparing normal progenitors. Blood Cancer J.

[71] Crinier A, Dumas PY, Escaliere B, Piperoglou C, Gil L, Villacreces A, et al. Single-cell profiling reveals the trajectories of natural killer cell differentiation in bone marrow and a stress signature induced by acute myeloid leukemia. Cell Mol Immunol 2020 https://doi.org/10.1038/s41423-020-00574-8, 18, 5, 1290, 1304. 10.1038/s41423-020-00574-8 PMC8093261 33239726

